# Short-Term Oral UMP/UR Administration Regulates Lipid Metabolism in Early-Weaned Piglets

**DOI:** 10.3390/ani9090610

**Published:** 2019-08-27

**Authors:** Yumei Zhang, Songge Guo, Chunyan Xie, Ruxia Wang, Yan Zhang, Xihong Zhou, Xin Wu

**Affiliations:** 1 Key Laboratory of Agro-ecological Processes in Subtropical Region, Institute of Subtropical Agriculture, Chinese Academy of Sciences; National Engineering Laboratory for Pollution Control and Waste Utilization in Livestock and Poultry Production; Hunan Provincial Engineering Research Center for Healthy Livestock and Poultry Production; Scientific Observing and Experimental Station of Animal Nutrition and Feed Science in South-Central, Ministry of Agriculture, Changsha 410125, China; 2 College of Bioscience and Biotechnology, Hunan Agricultural University, Changsha 410128, Hunan, China; 3 Institute of Biological Resources, Jiangxi Academy of Sciences, Nanchang 330096, China; 4 Meiya Hai’an pharmaceutical Co., Ltd., Hai’an 226600, China

**Keywords:** Uridine monophosphate, Uridine, Lipid metabolism, Early-weaned piglets

## Abstract

**Simple Summary:**

Uridine monophosphate (UMP) and uridine (UR) are rich in sow’s milk. The results from this study showed that UMP and UR affect the lipid profile and lipid metabolism in weanling piglets. It is suggested that UMP and UR improve the energy status in early-weaned piglets.

**Abstract:**

As a main ingredient of milk, the nucleotides content is about 12–58 mg/g, which plays a critical role in maintaining cellular function and lipid metabolism. This study was conducted to evaluate the effects of short-term uridine monophosphate (UMP) and uridine (UR) administration on lipid metabolism in early-weaned piglets. Twenty-one weaned piglets (7 d of age; 3.32 ± 0.20 kg average body weight) were randomly assigned into three groups: The control (CON), UMP, and UR group, and oral administered UMP or UR for 10 days, respectively. The results showed that supplementation with UMP significantly increased (*p <* 0.05) serum low density lipoprotein (LDL) and tended to increase (*p =* 0.062) serum total cholesterol (TC) content of piglets when compared with the other two groups. Oral administration with UMP and UR significantly decreased (*p* < 0.05) the serum total bile acid (TBA) and plasma free fatty acids (FFA) of piglets, and significantly reduced the fatty acid content of C12:0 (*p* < 0.01) and C14:0 (*p* < 0.05) in liver. Experiments about key enzymes that are involved in *de novo* synthesis of fatty acid showed that the gene expression of liver X receptors (*LXRα*), sterol regulatory element-binding transcription factor 1 (*SREBP1c*), fatty acid desaturase 2 (*FADS2*), and fatty acid elongase 5 (*ELOVL5*) were remarkably down-regulated (*p* < 0.05) with UMP and UR treatment, and key factors of adipose triglyceride lipase (*ATGL*), hormone-sensitive lipase (*HSL*), and carnitine palmitoyl transferase 1 (*CPT-1α*) involved in fatty acid catabolism were also decreased (*p* < 0.05). Additionally, the protein expression of phosphorylated-mTOR was not affected while phosphorylation of AKT was repressed (*p* < 0.05). In conclusion, short-term oral UMP or UR administration could regulate fatty acid composition and lipid metabolism, thus providing energy for early-weaned piglets.

## 1. Introduction

Weaning has been reported to reduce the digestion and absorption of nutrients, thereby reducing feed intake and growth performance [1,2]. Dietary exogenous growth factors, as important energy sources after weaning, have been widely studied for many years [1,3]. Among others, exogenous nucleotides are a group of biologically active substances that play key roles in most of the biological processes, especially in the fast growing period with limited nutrient conditions [4,5]. Previous research demonstrated that nucleotides are abundant in mammal’s milk, and the content of uridine 5’-monophosphate (5’-UMP) accounts for 98% of all 5’ monophosphate nucleotides in sow colostrum, which showed a serious decline from 0 to 28 days [6,7], indicating the high demand for nucleotides in early-weaned piglets. Uridine (UR), which is a metabolic product of UMP, plays a critical role in maintaining cellular function, lipid metabolism, and energy homeostasis [8,9,10]. What is more, our previous studies have shown that exogenous UMP/UR supplementation improve the growth performance (average daily feed intake and average daily gain) [11] and promote intestinal development [1] and nucleotide transport [5] of weaned piglets.

Recent research has shown that UR can prevent fenofibrate-induced fatty liver via regulating lipid metabolism [8]. Most interestingly, short-term UR can prevent drug-induced liver lipid accumulation, while long-term UR treatment showed an opposite effect and induced serious liver lipid accumulation in mice [8,12]. However, the specific mechanism has not been elaborated and there is no information regarding the regulation of UMP/UR on lipid metabolism in animal nutrition. Hence, we made the working hypothesis that short-term oral UMP and UR administration can provide energy for early-weaned piglets by regulating lipid metabolism. 

## 2. Materials and Methods 

### 2.1. Ethics Statement

The Protocol Management and Review Committee of the Institute of Subtropical Agriculture at the Chinese Academy of Science approved animal experiments. Piglets were cared for and slaughtered in accordance with the guidelines of the institute of Subtropical Agriculture on Animal Care (Changsha, China), with protocol 2015-8A.

### 2.2. Animals and Experimental Design

Twenty-one crossbred piglets (Duroc × Landrace × Yorkshire) weaned at seven days were randomly assigned into three experimental groups according to the average body weight (BW = 3.32 ± 0.20 kg, n = 7): control group, supplemented with water 4 mL/d; UMP group, supplemented with 0.119 g/mL UMP 4 mL/d; and, UR group, supplemented with 0.087 g/mL uridine 4 mL/d. Water, UMP, and UR were orally administered while using a disposable syringe at 8:00 am once a day. The piglets were housed in individual pens and fed the diets ad libitum for 10 days. UMP and UR were obtained from Meiya Hai’an pharmaceutical Co., Ltd. (Hangzhou, China) and the purity is 99.80% and 99.90%, respectively. The dose of UMP and UR was determined by the average content of uridylic acid in sow milk with equivalent conversion and the diet of piglets was based on milk powder (meeting the NRC 2012 piglet nutrition standards). The composition and nutritional levels of the diets were composed as indicated in Table 1.

### 2.3. Samples Collection 

The blood samples were collected from the jugular vein into two tubes (heparinized tubes and non-heparinized tubes) from fasted piglets at the end of the trial [13]. The blood samples were then centrifuged at 3000 g for 15 min. at 4 °C to obtain plasma and serum samples and stored at –80 °C for subsequent biochemical analysis [14]. Thereafter, following euthanasia, the liver samples were immediately snap frozen in liquid nitrogen and then stored at −80 °C for further analysis [15].

### 2.4. Serum Biochemical Indices

An Automated Biochemistry Analyzer (BS-190, Mindray, Shenzhen, China) was used to analyse the concentrations of serum triglyceride (TG), total cholesterol (TC), glucose (Glu), low density lipoprotein (LDL), and total bile acid (TBA), according to the commercial kits (Nanjing Jiancheng Bioengineering Institute, Nanjing, China) [16].

### 2.5. Plasma Free Fatty Acids 

The plasma samples were thawed at 4 °C and then free fatty acids (FFA) content were determined by a NEFA test kit (Wako Pure Chemical Industries, Ltd., Osaka, Japan), according to the manufacturer’s instructions.

### 2.6. Liver Fatty Acids 

The liver tissue samples were freeze-dried and ground. Subsequently, 0.5 g liver samples were mixed with 4 mL benzene-petroleum ether (v/v, 1/1) and the extracted lipids lasted for 24 h. FA methyl esters were prepared from liver samples via gas chromatography while using an Agilent 6890N Gas chromatograph (Agilent Technologies, Santa Clara, CA, USA), as described in our previous methods [17]. The fatty acids were quantified based on the peak area of each FA with an internal standard in the sample, and the concentrations were expressed as a percentage of total fatty acids present.

### 2.7. RNA Extraction and cDNA Synthesis 

Approximately 100 mg of liver sample were pulverized in liquid nitrogen and total RNA was isolated with TRIzol Reagent (Invitrogen, Carlsbad, CA, USA). The RNA quantity and quality were detected by ultraviolet spectroscopy while using a spectrophotometer (Nano Drop ND-1000; Thermo Fisher Scientific, New York, NK, USA), and RNA quality was considered to be an acceptable indicator when the OD 260/280 ratio is 1.8–2.0. Afterwards, each RNA sample was reverse-transcribed into cDNA while using Prime Script RT reagent Kit (Takara, Tokyo, Japan), according to the manufacturer’s instructions [18].

### 2.8. Real-Time Quantitative PCR (RT-qPCR)

Real-time PCR was performed using the primers that are shown in Table 2. Briefly, the reaction was run in Roche LightCycler^®^ 480II (Roche, Basel, Switzerland) with a total volume of 10 µL assay solution containing 5 µL SYBR Green mix (BioRad) (Thermo Fisher Scientific, New York, NY, USA), 2.4 µL nuclease-free water, 2 µL cDNA template, and 0.3 µL each of forward and reverse primers. After initial denaturation (10 min at 95 °C), 40 cycles of denaturation was conducted at 95 °C for 15 s, annealing for 30s, and extension for 60 s at 72 °C. This was followed by a melt curve analysis (with a rate of 0.1 °C/s from 70 °C to 95 °C) and fluorescence measurement [19]. The 2^−ΔΔCt^ method was used to quantitate mRNA expression relative to β-actin [20]. All of the genes were run in triplicate and the average values were reported. 

### 2.9. Total Protein Extraction and Western Blot Analysis

Liver tissues (n = 4) were homogenized in liquid nitrogen and the total proteins were extracted with RIPA buffer (Beyotime Biotechnology, Shanghai, China) containing 1 mM PMSF (KeyGEN BioTECH, Nanjing, China) and 1% phosphatase inhibitor. The samples were centrifuged at 12,000 g, 4 °C for 10 min., and the supernatants liquid was collected as protein samples. The protein content was determined by using BCA assay reagent (Beyotime Biotechnology, Shanghai, China) in accordance with the manufacturer’s instructions. All of the protein samples were adjusted to an equal concentration with 5× loading buffer (Beyotime Biotechnology, Shanghai, China) for protein denaturation [19]. Briefly, the total protein was isolated by 10% SDS-PAGE gel (Beyotime Biotechnology, Shanghai, China) and then transferred onto Immobilon^®^-P transfer membranes (Millipore Corp, Billerica, MA, USA). The membranes were blocked with 5% nonfat milk (Sangon Biotech (Shanghai) Co., Ltd., Shanghai, China), and then primary antibody against phospho-mTOR (Ser2448), mTOR, phospho-AKT (Ser473), AKT, and β-actin (Cell Signaling Technology Inc., Danvers, MA, USA) was applied overnight at 4 °C. After incubating with the secondary antibody at room temperature for 2 h, the membrane was detected by chemiluminescence (Applygen Technologies Inc., Beijing, China) [21].

### 2.10. Statistical Analysis

All the data were analyzed by one-way ANOVA, followed Duncan’s multiple-range test using the SPSS statistics 22 package (SPSS Institute, Inc., Chicago, IL, USA). Data were presented as means ± standard error of the mean (SEM), and considered to be statistically significant at *p* < 0.05, while a trend was considered significant at 0.05 ≤ *p* ≤ 0.10.

## 3. Results

### 3.1. Serum Biochemical Indices

Table 3 shows the serum biochemical indices of weaned piglets. In this study, UMP oral supplemented significantly increased (*p* < 0.05) the concentration of serum LDL, and tended to increase (*p =* 0.062) serum TC content of piglets when compared to those in the other two groups. Piglets oral administration of UMP and UR significantly decreased (*p* < 0.05) the content of TBA in serum. However, no significant differences were found in serum TG and Glu indices between the treatment and CON group (*p* > 0.10).

### 3.2. Plasma Free Fatty Acid

**Figure 1:**
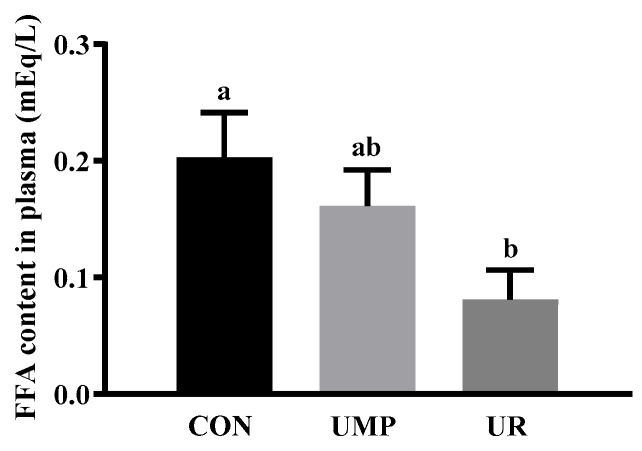
The content of plasma free fatty acid in the piglets. FFA, free fatty acids. Values are means (n = 7) with their standard errors represented by vertical bars, a, b means values with different letters were significantly different (*p* < 0.05).

The plasma free fatty acids (FFA) were significantly decreased (*p* < 0.05) in piglets from the UR group when compared to the CON group (Figure 1).

### 3.3. Liver Fatty Acid Profile

The liver fatty acid profiles of the piglets are shown in Table 4. Interestingly, it was found that dietary UMP and UR oral supplementation significantly reduced the content of C12:0 fatty acid (*p* < 0.01) and C14:0 fatty acid (*p* < 0.05) in liver. There were no treatment differences in other lipid profiles (*p* > 0.10). 

### 3.4. Relative Gene Expression of Lipid Metabolism

**Figure 2:**
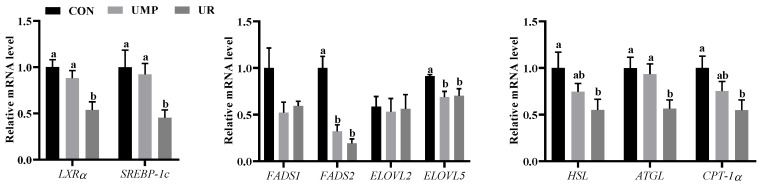
mRNA expression of genes involved in fatty ac**i**d metabolism in the liver. *LXRα*, liver X receptors; *SREBP1c*, sterol regulatory element-binding transcription factor 1; *FADS1*, fatty acid desaturase 1; *FADS2*, fatty acid desaturase 2; *ELOVL2*, fatty acid elongase 2; *ELOVL5*, fatty acid elongase 5; *HSL*, hormone-sensitive lipase; *ATGL*, adipose triglyceride lipase; *CPT-1α*, carnitine palmitoyl transferase 1. Values are means (n = 7) with their standard errors represented by vertical bars, a, b means values with different letters were significantly different (*p* < 0.05).

Figure 2 shows the mRNA expression of lipid metabolism in liver. When compared with the control group, the mRNA expression of liver X receptors (*LXRα*) and sterol regulatory element-binding transcription factor 1 (*SREBP1c*) showed a significant decrease (*p* < 0.05) in UR group, the mRNA expression of fatty acid desaturase 2 (*FADS2*) and fatty acid elongase 5 (*ELOVL5*) were significantly lower (*p* < 0.05) in the UMP and UR groups. The relative levels of hormone-sensitive lipase (*HSL*), adipose triglyceride lipase (*ATGL*), and carnitine palmitoyl transferase 1 (*CPT-1α*) were down-regulated in UR group as compared with the CON group (*p* < 0.05).

### 3.5. Expression of Proteins Involved in Energy Metabolism

**Figure 3:**
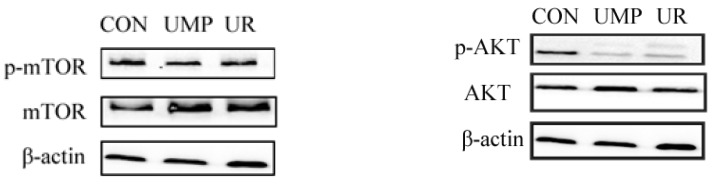

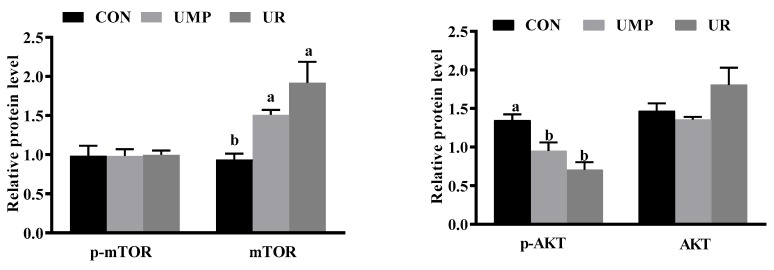
Relative protein expressions of mTOR and AKT in the liver of piglets were assesses by immunoblot analysis. mTOR, mammalian target of rapamycin; AKT, also known as Protein Kinase B. Protein expression levels were normalized using β-actin. Values of different letters (a, b) differ (*p* < 0.05). Values are the mean ± SEM (n = 4).

Dietary UMP and UR oral supplementation did not affect the protein expressions of phosphorylated-mTOR (*p* > 0.05, Figure 3), while markedly decreased the abundance of phosphorylated-AKT in the liver (*p* < 0.05).

## 4. Discussion

Nucleotides and their derivatives play key roles in most of the biological processes, such as maintaining cellular function, nucleic acids metabolism, and lipid metabolism [8,22,23]. Nucleotides are considered to be nonessential nutrients. However, under special conditions, especially in the case of disease, rapid growth, and immune challenge, exogenous nucleotides become essential [4]. Our previous study also showed that supplementation with exogenous nucleotides could improve the average daily gain (ADG) in early weaned piglets [11]. As mentioned earlier, UMP accounts for a large proportion of the nucleotides present in porcine milk, and exogenous nucleotide have been shown to regulate lipid metabolism [6,8]. Thus, we try to explain whether dietary UMP and UR supplementation could increase ADG of piglets via affecting lipids metabolism. The lipid profiles are affected by the metabolism of related lipoproteins, including particles that transport water-insoluble lipids and cholesterol in the blood circulation [24]. Low density lipoprotein (LDL), as the main component of serum total cholesterol, is related to risk of atherosclerotic cardiovascular disease (CVD) [25], responsible for carrying cholesterol and triglycerides from liver to peripheral tissues [26]. Le et al. reported that uridine supplementation had no effects on blood cholesterol and LDL in 10–12 weeks old C57bl/6 mice [8]. The results of the current study showed that oral supplementation with uridine showed no effects on TC and LDL levels in serum, which are consistent with these reports. However, UMP supplementation significantly increased the LDL levels and numerically increased the TC content of serum; a possible explanation is that short-term supplementation with UMP promotes the ability of LDL to carry cholesterol. Total bile acids assist in the absorption of food cholesterol and is the final product of cholesterol metabolism in liver [27,28]. Our results showed that UMP and UR administration significantly decreased the serum total bile acid concentration, suggesting that short-term UMP and UR administration may accelerate cholesterol excretion or decrease the synthesis of bile acids, whether with few or no side effects need to be further discovered.

Glucose circulates into tissue cells through the bloodstream, providing energy for life activities [29]. A previous study also showed that uridine injection could increase the levels of uridine diphosphonate (UDP)-glucose in skeletal muscle [30]. In the present study, the plasma glucose did not differ among the experimental groups, which indicated that the ability of glucose supplying energy remained stable in the UR and UMP groups.

Early weaned piglets are characterized by physiological changes that lead to a decrease of feed intake and energy deficiency [31,32]. FFA were regarded as an important energy sources for various organs and tissues, especially during the early weaning period. The plasma content of FFA are regulated both by adipose tissues mobilization and utilization in various organs and tissues [4]. The decreased plasma FFA levels in the UR group indicated that FFA were probably utilized for providing energy to the piglets. As important substrates for the energy metabolism [33], medium-chain fatty acids (MCFAs) are abundant in milk lipids of mammals [34,35]. It has been reported that uridine supplementation reduced liver LCFAs accumulation in fenofibrate-induced fatty liver mice [8]. Interestingly, in the current study, UMP and UR oral administration also decreased the content of lauric acid (C12:0) and myristic acid (C14:0) in the liver of piglets, which indicated that short-term UMP and UR administration might stimulate the energy supplement of MCFAs.

Liver, as an important tissue for the biosynthesis of nucleotide, plays a critical role in both glucose and lipid metabolism [8,36]. As mentioned earlier, uridine in the diet can modulate liver lipid metabolism [22], and the expression of genes that are involved in lipid metabolism was consistent with the liver fatty acids profile in piglets [37]. Moreover, lipid metabolism involves *de novo* synthesis and the degradation of fatty acids [14]. The polyunsaturated fatty acids (PUFAs) act as modulators of the *LXRs* and *SREBPs* to further stimulate the *de novo* synthesis of fatty acids [38]. In our results, the mRNA expression of genes that are involved in *de novo* lipogenesis (*LXRα* and *SREBP-1c*) was decreased in the UR group when compared with the CON and UMP group, which was consistent with the plasma fatty acid level and liver MCFA content. This suggests that oral supplementation with UR decreased the *de novo* synthesis of lipids. Further research needs to be done to explore the roles of *SREBP1c* and *LXRα* on the biosynthesis of PUFAs. The *de novo* synthesis site of PUFAs in liver is catalyzed by two key enzymes, the fatty acid desaturases (*FADS*) and fatty acid elongase (*ELOVL*) [14]. *FADS2* catalyze the introduction of a double bond at the delta 6 positions [14], and *ELOVL5* is involved in the elongation of the C18-C20 PUFAs [39]. More recently, it has been found that the up-regulation of *ELOVL5* expression is primarily directly regulated by *SREBP-1c* [40]. The present study showed that the oral administration of UR and UMP down-regulated the expression of *ELOVL5*, as the same with the expression of *SREBP-1c*, suggested that there was a lower rate of *de novo* synthesis of PUFA in liver. Combined with the previous results, the content of C12:0 fatty acid and C14:0 fatty acid in liver was significantly reduced in the UR and UMP groups, which indicates that fatty acids might being used for providing energy in piglets. Lipolysis as the biochemical pathway responsible for the catabolism of triacylglycerols (*TAGs*) stored in the cellular lipid droplets, which degrades TG and releases FFA and glycerol into the circulation [41]. The major enzymes that are involved in the catabolism of *TAGs* are *ATGL* and *HSL* [42]. The present study showed that the mRNA expression of *ATGL* and *HSL* in the UR group was lower than the other two groups, which was consistent with the low FFA content of plasma. This suggests that oral supplementation with uridine decreased lipolysis in liver. In animal cells, another major pathway for fatty acid catabolism is β-oxidation that is catalyzed by *CPT-1* [43]. The lower expression of *CPT-1α* in the UMP and UR groups indicated the low β-oxidation activity of long-chain fatty acids, which indicated that UMP and UR decreased lipid degradation, as consistent with previous results.

The mTOR Complex 1 (mTORC1) regulates many processes that are related to the growth, including protein synthesis, autophagy, and lipogenesis, which can also promote the function of SREBP [44]. We found that UMP and UR treatment could improve the total protein expression of mTOR while with no influence on the phosphorylation of mTOR in the liver, which indicates that UMP and UR regulate lipogenesis through an mTOR independent pathway. Interestingly, we found that the protein expression of phosphorylation of AKT was decreased in the UMP and UR groups, which can stimulate *de novo* lipid synthesis through activating SREBP isoforms and was shown to be reduced with long-term uridine administration [12,36]. This suggests that short-term oral supplementation with UMP and UR stimulate *de novo* lipid synthesis may via AKT, which further regulated the expression of hepatic SREBP1c and provided energy for early-weaned piglets. However, the molecular mechanism about AKT regulating SREBP1C expression remains to be further explored.

## 5. Conclusion

The results in the present study showed that short-term UMP and UR treatment might stimulate *de novo* lipid synthesis via the AKT pathways to affect fatty acid content, thereby providing energy for early-weaned piglets. It would provide new insights into the role of the supplementation of dietary nucleotides as well as midchain fatty acids in weaned piglets.

## Figures and Tables

**Table 1:** Ingredient and nutrient levels of basal milk (air-dry basis).

Ingredient Composition	(%)	Nutrient Levels	(%)
Skimmed milk powder	85.0	DE ^a^ (MJ/kg)	14.65
Dried whey	5.0	CP ^b^	20.50
Glucose	2.5	Calcium	0.70
Plasma proteins	3.5	Total phosphorus	0.60
Premix *	4.0	Lysine	1.45
Total	100.0	Methionine	0.48
		Tryptophan	0.29

* The premix provided the following per kg of diets: VA1, 500 IU; VD3, 200 IU; VE, 85 IU; D-pantothenic acid, 35 mg; VB2, 12 mg; folic acid, 1.5 mg; nicotinic acid, 35 mg; VB, 13.5 mg; VB6, 2.5 mg; biotin, 0.2 mg; VB12, 0.05 mg; Cu (as copper sulfate), 15 mg; Fe (as ferrous sulfate), 100 mg; Mn (as manganese sulfate), 20 mg; I (as calcium iodate), 1.0 mg; Se (as sodium selenite), 0.35 mg; Co (as cobalt sulfate), 0.2 mg; Chr (as chromium Picolinate), 0.2 mg. ^a^ DE = digestible energy; ^b^ CP = Crude protein.

**Table 2:** Primers used for real-time PCR.

Gene	Accession No.	Nucleotide Sequence of Primers (5’-3′)	Product Size (bp)
*LXRα*	NM_001101814.1	F: GTAGATGGCTGAGGCGTGAC	96
		R: TTCCCAACCCTTTGACTCTTT	
*SREBP1c*	NM_214157.1	F: CCTCTGTCTCTCCTGCAACC	229
		R: GACCGGCTCTCCATAGACAA	
*ELOVL2*	XM_013977421.1	F: ATTCTTCACCACCAGCGAGG	131
		R: TGCCTGGCTGTTATCACTCG	
*ELOVL5*	XM_013986754.1	F: TACCACCATGCCACTATGCT	102
		R: GACGTGGATGAAGCTGTTGA	
*CPT-1α*	NM_001129805.1	F: CCATCAAAACTGCCTTCCTTAG	118
		R: AGCGAGTGTGCCAGATACAAA	
*FADS1*	NM_001113041.1	F: GTCACTGCCTGGCTCATTCT	155
		R: AGGTGGTTCCACGTAGAGGT	
*FADS2*	NM_001171750.1	F: ACGGCCTTCATCCTTGCTAC	144
		R: GTTGGCAGAGGCACCCTTTA	
*ATGL*	NM_001098605.1	F: ATGGTGCCCTACACGCTG	111
		R: GCCTGTCTGCTCCTTTATCC	
*HSL*	HM591297.1	F: GAAGGGAGAGCTATGGCACC	130
		R: CTCACACTCTCCAAGCCCAG	
*β-actin*	XM_003357928.2	F: CGTTGGCTGGTTGAGAATC	132
		R: CGGCAAGACAGAAATGACAA	

**Table 3:** Effect of uridine monophosphate (UMP) and uridine (UR) on serum biochemical indices of piglets (n = 7).

Items	CON	UMP	UR	SEM	*p*-Value
TG (mmol/L)	0.357	0.337	0.341	0.219	0.933
TC (mmol/L)	2.346 ^ab^	2.630 ^a^	2.114 ^b^	0.092	0.062
Glu (mmol/L)	5.359	4.577	4.553	0.207	0.193
LDL (mmol/L)	0.801 ^b^	1.049 ^a^	0.790 ^b^	0.048	0.039
TBA (umol/L)	18.050 ^a^	12.271 ^b^	7.317 ^c^	1.493	0.008

TG, serum triglyceride; TC, total cholesterol; Glu, glucose; LDL, low density lipoprotein; TBA, total bile acid. Values within a row with different superscripts (a, b) differ significantly (*p* < 0.05).

**Table 4:** Effect of UMP and UR on liver lipid profile in piglets (n = 7).

Fatty Acid Composition, %	CON	UMP	UR	SEM	*p*-Value
C12:0	0.195 ^a^	0.116 ^b^	0.095 ^b^	0.015	0.007
C14:0	0.788 ^a^	0.603 ^b^	0.578 ^b^	0.036	0.031
C16:0	15.340	15.507	15.121	0.226	0.793
C17:0	0.398	0.411	0.395	0.013	0.865
C18:0	21.237	21.656	21.922	0.276	0.631
C16:1	0.907	0.768	0.760	0.095	0.802
C18:1n9c	17.734	17.846	17.856	0.380	0.991
C20:1	0.313	0.302	0.300	0.012	0.903
C18:2n6c	15.954	16.406	16.196	0.200	0.686
C20:3n6	2.449	2.297	2.675	0.129	0.491
C20:4n6	17.668	17.623	17.459	0.239	0.938
C22:6n6	6.401	5.923	6.043	0.138	0.375
SFA	38.108	38.438	38.273	0.201	0.821
MUFA	19.080	19.006	19.034	0.395	0.997
PUFA	42.812	42.556	42.681	0.291	0.945
EPA	16.123	16.543	16.338	0.200	0.722

SFA, saturated fatty acid; MUFA, monounsaturated fatty acids; PUFA, polyunsaturated fatty acids; EPA, eicosapntemacnioc acid. Values within a row with different superscripts (a, b) differ significantly (*p* < 0.05).
